# TEP versus TAPP: comparison of the perioperative outcome in 17,587 patients with a primary unilateral inguinal hernia

**DOI:** 10.1007/s00464-015-4150-9

**Published:** 2015-03-25

**Authors:** F. Köckerling, R. Bittner, D. A. Jacob, L. Seidelmann, T. Keller, D. Adolf, B. Kraft, A. Kuthe

**Affiliations:** 1 Department of Surgery and Center for Minimally Invasive Surgery, Academic Teaching Hospital of Charité Medical School, Vivantes Hospital, Neue Bergstraße 6, 13585 Berlin, Germany; 2 Hernia Center, Winghofer Medicum, Winghofer Straße 42, 72108 Rottenburg am Neckar, Germany; 3 StatConsult GmbH, Halberstädter Straße 40 a, 39112 Magdeburg, Germany; 4 Department of General and Visceral Surgery, Diakonie Hospital, Rosenbergstraße 38, 70176 Stuttgart, Germany; 5 Department of General and Visceral Surgery, German Red Cross Hospital, Lützerodestraße 1, 30161 Hannover, Germany

**Keywords:** TEP, TAPP, Intraoperative complications, Postoperative complications, Inguinal hernia repair, Seroma

## Abstract

**Introduction:**

More than 20 years since the introduction of TAPP and TEP into clinical routine, there is a lack of clarity due to conflicting comparative data. Therefore, more results from registries are needed.

**Patients and methods:**

A total of 17,587 patients were enrolled prospectively between September 1, 2009, and April 15, 2013, in the Herniamed registry. Of these patients, 10,887 (61.9 %) had a TAPP and 6700 (38.1 %) a TEP repair. The dependent variables were intra- and postoperative complication rates, number of reoperations as well as absolute and relative frequencies. The results of unadjusted analyses were verified via multivariable analyses.

**Results:**

Multivariable analysis verified the results of unadjusted analysis, indicating that the surgical technique did not have any significant impact, also while taking account of other factors, on occurrence of intraoperative [*p* = 0.1648; OR = 1.214 (0.923; 1.596)] and general postoperative complications [*p* = 0.0738; OR = 1.315 (0.974; 1.775)]. Postoperative surgical complications [OR = 2.323 (1.882; 2.866); *p* < 0.0001] were noted more often after TAPP. Furthermore, the hernia defect size [*p* < 0.0001; I vs III: OR = 0.439 (0.313; 0.615), II vs III: OR = 0.712 (0.582; 0.872)] or scrotal [*p* < 0.0001; OR = 2.170 (1.501; 3.137)] hernia and age [*p* = 0.0002; 10-year OR = 1.135 (1.062; 1.213)] had a significant impact on the occurrence of postoperative complications. Complications were observed more commonly for larger hernia defects and a scrotal hernia. However, the difference in the postoperative complication rate between TEP and TAPP did not result in any difference in the reoperation rate (TEP 0.82 % vs TAPP 0.90 %; *p* = 0.6165).

**Conclusion:**

The intraoperative and general postoperative complication rates as well as the reoperation rate for complications show no significant difference between TEP and TAPP. The higher postoperative complication rate for TAPP, which could be managed conservatively, is partly explained by larger defect sizes, more scrotal hernias and older age.

Already 20 years ago, the first study was published comparing the two minimally invasive surgical techniques—transabdominal preperitoneal patch plasty (TAPP) versus total extraperitoneal patch plasty (TEP)—for surgical repair of inguinal hernia [1]. Since then, 23 further studies [2–24] have been published, including four systematic reviews and meta-analyses [2–5], 10 randomized controlled [6–15], two population-based [16, 17] and six case–control and observational studies [18–24]. The findings are contradictory. Due to the confusing data situation, all four systematic reviews/meta-analyses concluded that operation-related results for TEP and TAPP were similar, and the superiority of one method over the other could not be demonstrated and further studies were needed. The reason for this lack of clarity, which has now persisted for more than 20 years since the introduction of both techniques into clinical routine practices, is due in particular to the limited quality of the studies conducted so far. For example, only in two [11, 15] of the randomized controlled trials (RCTs) was comparison of TAPP with TEP the primary endpoint. In the eight remaining studies, the main focus was on comparison of laparoscopic with open surgery. Another critical aspect was the small number of patients per group, which did not exceed 30 in the six RCTs [6–9, 11, 12]. Besides, a duration of operation of more than 100 min [14] as well as a 25 % recurrence rate [6] suggests that the surgeons had not yet mastered the learning curve. Lack of experience must be viewed as being one of the main confounders as regards the results obtained. The study that must be deemed to be the best so far is the Swiss Registry Study published in 2012 [16]. Based on prospective data on 4552 patients undergoing TEP (*n* = 3457) and TAPP (*n* = 1095) of the Swiss Association of Laparoscopic and Thoracoscopic, both techniques were found to have a low complication rate, with that of TAPP being the lower of the two. Likewise, the duration of operation was shorter, but the length of hospital stay was half a day longer than for TEP. The reasons for these differences were not discussed. TAPP and TEP differ only in terms of the access route, and the inguinal surgical method is similar. If differences are found between the two techniques, these are due either to the use of a different access route, other hernia disease, or to variation of experience among surgeons.

On the basis of prospective data from the German hernia registry Herniamed collected for a very large patient group data in everyday routine practice, we now explore whether differences can also be discerned in the perioperative outcome between TEP and TAPP and what the likely reasons for these are.

## Patients and methods

The Herniamed quality assurance study is a multicenter, internet-based hernia registry [25] into which 358 participating hospitals and surgeons engaged in private practice (Herniamed Study Group) in Germany, Austria and Switzerland (status: April 2013) had entered data prospectively on their patients who had undergone hernia surgery. All postoperative complications occurring up to 30 days after surgery are recorded. On 1-year follow-up, postoperative complications are once again reviewed when the general practitioner and patient complete a questionnaire. This present analysis compares the prospective data collected for all patients who had undergone primary unilateral inguinal hernia repair using either transabdominal preperitoneal patch plasty (TAPP) or total extraperitoneal patch plasty (TEP). Inclusion criteria were minimum age of 16 years and primary unilateral inguinal hernia. There were no exclusion criteria used in this study beyond those who fell out of the inclusion criteria. In total, 17,587 patients were enrolled between September 1, 2009, and April 15, 2013. Of these patients, 10,887 (61.9 %) had a TAPP repair and 6700 (38.1 %) a TEP repair.

The demographic and surgery-related parameters included age (years), sex (m/f), ASA classification (I–IV) as well as the proportion of scrotal inguinal hernias and the hernia defect size based on EHS classification (Hernia type: medial, lateral, femoral, scrotal. Defect size: Grade I = <1.5 cm, Grade II 1.5–3 cm, Grade III >3 cm) [26, 27]. The dependent variables were intra- and postoperative complication rates, number of reoperations as well as absolute and relative frequencies; continuous variables are displayed as mean, median, standard deviation and ranges.

All analyses were performed with the software SAS 9.2 (SAS Institute Inc. Cary, NY, USA) and deliberately reviewed to the full level of significance. Each *p* value ≤0.05 thus represents a statistically significant result. To discern differences between the groups in unadjusted analyses, Fisher’s exact test was used for categorical outcome variables, and the *t* test for continuous variables. For data that did not follow the normal distribution, as in the case of duration of operation and length of stay, the distribution was first transformed with the natural logarithm.

To rule out any confounding of data caused by different patient characteristics, the results of unadjusted analyses were verified via multivariable analyses in which, in addition to operation technique, other influence parameters were simultaneously reviewed.

To access influence factors in multivariable analyses, the general linear model was used for continuous outcome variables, and the binary logistic regression model for dichotomous outcome variables. Estimates for odds ratio (OR) or least square (LS) means, respectively, and the corresponding 95 % confidence interval were given. For age [years], the 10-year OR estimate was given. Results are presented in tabular form, sorted by descending impact. Patients (and not hernia) were the level of analysis.

## Results

### Unadjusted analysis

The patients in the TEP and TAPP groups did not differ in terms of age or gender distribution. However, there were significant differences between the two patient groups in respect of a number of other patient characteristics. Table 1 shows the overall demographic data. No difference was found with regard to age or gender distribution. However, more patients with a lower ASA status and larger hernia defects underwent the TAPP method. The TAPP technique was also used more often for hernias with ‘medial,’ ‘scrotal’ and ‘combined’ localization, while the TEP technique was employed more commonly for lateral hernias.

**Table 1:** Demographic and surgery-related parameters

	TEP	TAPP	*p*
*Demographic parameters*			
Age			
Years ± SD	55.04 ± 15.95	55.40 ± 15.71	0.1441
Range	16–100	16–98	
Sex			
Male	5862 (87.49 %)	9441 (86.72 %)	
Female	838 (12.51 %)	1446 (13.28 %)	0.1394
ASA score			
I	2206 (32.93 %)	3831 (35.19 %)	
II	3624 (54.09 %)	5725 (52.59 %)	
III	851 (12.7 %)	1313 (12.06 %)	
IV	19 (0.28 %)	18 (0.17 %)	0.0071
*Surgery-related parameters*			
Hernia type			
Medial	2057 (30.7 %)	4188 (38.47 %)	<0.0001
Lateral	5274 (78.72 %)	7364 (67.64)	<0.0001
Femoral	256 (3.82 %)	479 (4.4 %)	0.0627
Scrotal	132 (1.97 %)	325 (2.99 %)	<0.0001
Defect size			
I (<1.5 cm)	1336 (19.94 %)	1852 (17.01 %)	
II (1.5–3 cm)	4094 (61.1 %)	6901 (63.39 %)	
III (>3 cm)	1270 (18.96 %)	2134 (19.6 %)	<0.0001

As regards the outcome variables, the two surgical methods differed in terms of duration of operation (<0.0001) and of postoperative length of hospital stay (<0.0001). Both were significantly longer for patients in the TAPP group. The mean duration of operation for the TAPP technique was 52.62 ± 23.58 min, and the median was 47 min (range 20–274 min). The mean duration of operation for the TEP technique at 48.58 ± 21.52 min and median at 45 min (range 20–275 min) was significantly lower (Table 2).

**Table 2:** Duration of operation. Length of stay and unadjusted *p* values

	Mean	SD	Min	Max	Median	*p*
Duration of operation (min)						
TEP	48.53	21.52	20	275	45	<0.0001
TAPP	52.62	23.58	20	274	47	
Length of stay (days)						
TEP	1.88	2.19	1	63	2	<0.0001
TAPP	1.93	2.22	1	64	2	

The mean length of hospital stay for the TAPP group patients was 1.93 ± 2.22 days, and for the TEP group patients, it was 1.88 ± 2.19 days (median in each case 2.0 days, range 1–63 days after TEP, 1–64 days after TAPP). Table 2 shows the total data for duration of operation and length of hospital stay.

Unadjusted analysis, at 1.19 % for TEP and 1.40 % for TAPP, did not reveal any significant differences in the intraoperative complications associated with the two surgical techniques (*p* = 0.2763).

Significantly, more complications were noted within the first 30 postsurgical days in the TAPP group (3.97 %; *p* < 0.0001). These were mainly due to the significant difference in the postoperative seroma rate (TEP 0.51 % vs TAPP 3.06 %; *p* < 0.001). Secondary bleeding occurred more frequently after TEP operation (1.15 %; *p* = 0.030), while seroma was seen more commonly after TAPP operation (3.06 %).

However, the difference in the postoperative complication rate between TEP and TAPP did not result in any difference in the reoperation rate due to surgical complications (TEP 0.82 % vs TAPP 0.90 %; *p* = 0.6165), i.e., the difference in the postoperative complication rate between TEP and TAPP referred only to postoperative complications that were amenable to conservative treatment. Early recurrences were not a reason for reoperation (Table 3).

**Table 3:** Intra- and postoperative complications and unadjusted *p* values

Unadjusted analysis	TEP	TAPP	*p*
Intraoperative complications	80 (1.19 %)	152 (1.40)	0.2763
Bleeding	53 (0.79 %)	108 (0.99 %)	0.1922
Injuries (total)	42 (0.63 %)	77 (0.71 %)	0.5705
Vascular	16 (0.24 %)	34 (0.31 %)	0.4662
Bladder	3 (0.04 %)	15 (0.14 %)	0.0867
Bowel	4 (0.06 %)	14 (0.13 %)	0.2256
Nerve	1 (0.01 %)	0 (0)	0.381
Postoperative complications	114 (1.70 %)	432 (3.97 %)	<0.0001
Bleeding	77 (1.15 %)	89 (0.82 %)	0.03
Intestinal lesion	0 (0)	4 (0.04 %)	0.3048
Impaired wound healing	9 (0.13 %)	10 (0.09 %)	0.4798
Seroma	34 (0.51 %)	333 (3.06 %)	<0.0001
Infection	3 (0.04 %)	4 (0.04 %)	1
Intestinal obstruction	0 (0)	6 (0.06 %)	0.0891
Reoperation	55 (0.82 %)	98 (0.90 %)	0.6165
General complications	65 (0.97 %)	137 (1.26 %)	0.0935
Fever	1 (0.01 %)	12 (0.11 %)	0.0228
Diarrhea	0 (0)	4 (0.04 %)	0.3048
Coronary heart disease	0 (0)	22 (0.20 %)	<0.0001
Exitus letalis	0 (0)	5 (0.05 %)	0.1641

Viewed in global terms, no significant differences were noted between the two groups as regards general complications. In terms of individual general complications, a significant difference was seen for fever (*p* = 0.0228) and coronary heart disease (*p* < 0.0001). Both occurred more commonly in patients operated on with the TAPP technique (0.11 vs 0.2 %, respectively). Table 3 illustrates all data related to complications.

Data to compare recurrence rates can be provided only later at the end of 1-year follow-up.

### Multivariable analysis

Multivariable analysis verified the results of unadjusted analysis, indicating that the surgical technique did not have any significant impact, also while taking account of other factors, on occurrence of intraoperative [*p* = 0.1648; OR = 1.214 (0.923; 1.596)] and general complications [*p* = 0.0738; OR = 1.315 (0.974; 1.775)]. The only variable impacting onset of intraoperative complications was medial inguinal hernia (*p* = 0.001). It had a preventive effect [OR = 0.607 (0.451; 0.816)] (Table 4). Onset of general complications was affected by a number of parameters, but not the surgical technique. A lower ASA score [*p* < 0.001, e.g., ASA III vs I: OR = 2.599 (1.645; 4.107)], younger age (10-year OR = 1.249 [1.116; 1.398], *p* = 0.0001) as well as medial [*p* = 0.03, OR = 0.577 (0.353; 0.942)] or lateral [*p* = 0.04; OR = 0.586 (0.352; 0.976)] inguinal hernia were preventive (Table 5), whereas a higher ASA score, older age and a scrotal hernia led to significantly more general postoperative complications.

**Table 4:** Multivariable analysis of intraoperative complications

Parameter	Category	Intraoperative complications	
		OR [95 % CI]	*p*
EHS medial	Yes versus no	0.607 [0.451; 0.816]	0.0001
OP method	TAPP versus TEP	1.214 [0.923; 1.596]	0.1648
Age*		1.061 [0.962; 1.170]	0.2339
Defect size	I (<1.5 cm) versus III (>3 cm)	0.950 [0.614; 1.470]	0.5775
	II (1.5–3 cm) versus III (>3 cm)	0.851 [0.614; 1.180]	
Sex	Male versus female	1.248 [0.819; 1.901]	0.3032
ASA	II versus I	1.059 [0.770; 1.455]	0.9110
	III versus I	1.189 [0.746; 1.897]	
	IV versus I		

* 10-year estimate

**Table 5:** Multivariable analysis of postoperative general complications

Parameter	Category	Postoperative general complications	
		OR [95 % CI]	*p*
ASA	II versus I	1.028 [0.699; 1.511]	<0.0001
	III versus I	2.599 [1.645; 4.107]	
	IV versus I	4.329 [0.970; 19.322]	
Age*		1.249 [1.116; 1.398]	0.0001
EHS medial	Yes versus no	0.577 [0.353; 0.942]	0.0279
EHS lateral	Yes versus no	0.586 [0.352; 0.976]	0.0401
OP method	TAPP versus TEP	1.315 [0.974; 1.775]	0.0738
Defect size	I (<1.5 cm) versus III (>3 cm)	1.232 [0.761; 1.996]	0.6148
	II (1.5–3 cm) versus III (>3 cm)	1.016 [0.713; 1.447]	
EHS femoral	Yes versus no	0.723 [0.327; 1.598]	0.4225
Sex	Male versus female	1.108 [0.709; 1.731]	0.6532
EHS scrotal	Yes versus no	0.912 [0.411; 2.027]	0.0001

* 10-year estimate

The significant influence exerted by the surgical technique on the postoperative complication rate also persisted after adjustment of the other influence variables. Postoperative complications [OR = 2.323 (1.882; 2.866); *p* < 0.0001] were noted more often after TAPP. For a postoperative complication rate of 3.1 %, this would amount to around 43 out of every 1000 patients operated on with TAPP and to 19 out of every 1000 patients operated on with the TEP technique. Furthermore, the hernia defect size [*p* < 0.0001; I vs III: OR = 0.439 (0.313; 0.615), II vs III: OR = 0.712 (0.582; 0.872)], presence of medial [*p* = 0.0007, OR = 0.610 (0.458; 0.811)], lateral [*p* = 0.0043; OR = 0.655 (0.490; 0.876)] or scrotal [*p* < 0.0001; OR = 2.170 (1.501; 3.137)] hernia and age [*p* = 0.0002; 10-year OR = 1.135 (1.062; 1.213)] had a significant impact on the occurrence of postoperative complications. Complications were observed more commonly for larger hernia defects and a scrotal hernia. Conversely, there were fewer postoperative complications in young patients and in patients with a medial or lateral hernia (Table 6).

**Table 6:** Multivariable analysis of postoperative surgical complications

Parameter	Category	Postoperative surgical complications	
		OR [95 % CI]	*p*
OP method	TAPP versus TEP	2.323 [1.882; 2.866]	<0.0001
Defect size	I (<1.5 cm) versus III (>3 cm)	0.439 [0.313; 0.615]	<0.0001
	II (1.5–3 cm) versus III (>3 cm)	0.712 [0.582; 0.872]	
EHS scrotal	Yes versus no	2.170 [1.501; 3.137]	<0.0001
Age*		1.135 [1.062; 1.213]	0.0002
EHS medial	Yes versus no	0.610 [0.458; 0.811]	0.0007
EHS lateral	Yes versus no	0.655 [0.490; 0.876]	0.0043
ASA	II versus I	1.097 [0.883; 1.363]	0.2321
	III versus I	1.135 [0.834; 1.545]	
	IV versus I	3.075 [1.023; 9.247]	
Sex	Male versus female	1.120 [0.832; 1.509]	0.4547
EHS femoral	Yes versus no	1.116 [0.700; 1.779]	0.6449

* 10-year estimate

Likewise, the multivariable model revealed the significant influence of the surgical technique on seroma formation or on secondary bleeding. For TAPP, postoperative seromas were seen significantly more often [OR = 5.873; (4.116; 8.380), *p* < 0.0001]. For every 1000 patients undergoing surgery, there would therefore be 35 seromas for TAPP patients compared with six seromas on using TEP. The presence of a scrotal inguinal hernia also had a significant effect on the seroma rate, with this being conducive to onset of seroma [*p* < 0.0001; OR = 2.784 (1.837; 4.217)]; smaller hernia defects [*p* = 0.0002; I vs III: OR = 0.398 (0.258; 0.615), II vs III: OR = 0.754 (0.590; 0.964)], a lateral [*p* = 0.001; OR = 0.566 (0.401; 0.799)] or medial inguinal hernia [*p* = 0.012; OR = 0.639 (0.451; 0.904)] each had a preventive effect, whereas older age [*p* = 0.003; 10-year OR = 1.131 (1.044; 1.226)] was conducive to onset of seroma.

For TAPP, secondary bleeding was less common [OR = 0.734 (0.539; 1.000), *p* = 0.05]. For a total secondary bleeding rate of 0.94 %, that complication would thus occur in eight out of every 1000 TAPP patients and in 11 out of every 1000 TEP patients. Conversely, the secondary bleeding rate was influenced more by the ASA status (*p* = 0.005), medial inguinal hernia (*p* = 0.02) and age (*p* = 0.04). A low ASA score, e.g., ASA III versus I: OR = 1.760 [1.038; 2.982], medial hernia [OR = 0.540 (0.323; 0.901)] and young age [10-year OR = 1.135 (1.007; 1.279), *p* = 0.0387] had a preventive effect on onset of secondary bleeding.

Multivariable analysis also confirmed that the surgical technique did not have any impact on the reoperation rate linked to complications. However, it was demonstrated that a high ASA classification as well as large hernia defects had a significant impact on the complication-related reoperation rate (Table 7).

**Table 7:** Multivariable analysis of reoperation

Parameter	Category	Reoperation	
		OR [95 % CI]	*p*
ASA	II versus I	1.074 [0.709; 1.627]	0.0153
	III versus I	1.845 [1.075; 3.166]	
	IV versus I	5.656 [1.232; 25.955]	
Defect size	I (<1.5 cm) versus III (>3 cm)	0.423 [0.227; 0.787]	0.0135
	II (1.5–3 cm) versus III (>3 cm)	0.658 [0.454; 0.954]	
EHS scrotal	Yes versus no	1.988 [1.007; 3.922]	0.0478
EHS medial	Yes versus no	0.720 [0.436; 1.188]	0.1986
Age*		1.054 [0.932; 1.192]	0.3990
OP method	TAPP versus TEP	1.097 [0.786; 1.533]	0.5855
EHS femoral	Yes versus no	0.849 [0.318; 2.262]	0.7431
EHS lateral	Yes versus no	0.918 [0.540; 1.560]	0.7528
Sex	Male versus female	1.009 [0.586; 1.735]	0.9754

* 10-year estimate

It was also possible to confirm the significant influence of the surgical technique on the duration of operation (*p* < 0.0001) and the postoperative length of hospital stay (*p* < 0.0001). The operation took longer for TAPP [49.74 min (47.76; 51.82), and was 45.86 min (44.00; 47.79) for TEP]. Other significant influence variables identified for the duration of operation were sex (*p* < 0.0001), ASA classification (*p* = 0.02), hernia defect size (*p* < 0.0001) and medial (*p* < 0.0001), femoral (*p* = 0.2) and scrotal (*p* < 0.0001) hernias. Die duration of operation was significantly longer for men, for patients with a lower ASA score, larger hernia defect or if surgery was performed for a femoral or scrotal hernia. Conversely, the duration of surgery was significantly shorter for a medial hernia.

In the multivariable model, too, the postoperative length of hospital stay was significantly longer for patients operated on with the TAPP technique [2.19 d (2.08; 2.31) for TAPP and 2.27 d (2.16; 2.39) for TEP]. Besides, other variables whose significant influence was confirmed were sex (*p* < 0.0001), ASA classification (*p* < 0.0001), hernia defect size (*p* = 0.0002), medial (*p* = 0.006), scrotal (*p* < 0.0001) or femoral (*p* = 0.02) hernia and also age (*p* < 0.0001). The length of hospital stay was prolonged for cases with higher ASA score or the presence of a scrotal or femoral hernia. It was also longer for rising age. The length of stay was shorter for a medial inguinal hernia or a smaller hernia defect. It was also significantly shorter for men.

## Discussion

This Registry study compared prospective data for 10,887 TAPP operations with 6700 TEP operations for primary unilateral inguinal hernia on the basis of the perioperative outcomes. These perioperative results were first investigated using unadjusted, and then multivariable, analysis for differences between TAPP and TEP, while identifying other influence variables. Thanks to the large number of cases, it was possible to identify the significant impact of even small differences, even if such an effect was not of clinical relevance.

The EHS classification for inguinal hernia has been used for the first time in the Herniamed Registry for precise stratification of the patient collective [25].

This makes it easier to identify variables impacting the perioperative outcome; it also makes it easier to identify patient characteristics as well as method-independent variables that affect the outcome.

For example, on the basis of the Herniamed data, no difference was seen in the age or gender distribution between the TEP and TAPP groups. Conversely, significant differences were discerned between the TEP and TAPP groups in terms of the proportion of medial, lateral and scrotal hernias. That also applied for the defect size. Significantly, more medial and scrotal hernias as well as larger defects were seen for the TAPP group. Despite that disparity, no difference was seen in the intraoperative complication rate between TEP and TAPP. The significant difference in the postoperative complication rates, which were higher for TAPP (TEP 1.70 vs TAPP 3.97; *p* < 0.0001), was due to a significantly higher seroma rate (TEP 0.51 % vs TAPP 3.06 %; *p* < 0.0001). In multivariable analysis, the variables identified as impacting onset of a postoperative complication, in particular seroma formation, were a large hernia defect and a scrotal hernia. Both hernia pathologies were found significantly more often in patients operated on with the TAPP technique, hence this higher complication rate compared with TEP was observed across different patient collectives. However, despite adjustment of these parameters, TAPP per se proved to be a lower but independent risk factor. To what extent the various surgeon’s experience played a role here cannot be elucidated on the basis of that analysis. Since there is a greater number of TAPP surgeons in the Herniamed Registry, it is presumed that TEP tends to be performed more by specialists.

Nonetheless, the significant difference in the postoperative complication rates did not give rise to a significant difference in the complication-related reoperation rates between TEP and TAPP. Hence, the implicated complications were essentially postoperative complications that were amenable to conservative treatment in the TAPP group.

As such, the difference in perioperative outcome between TEP and TAPP must be imputed more to the indication than to the surgical technique. Since a greater number of large inguinal hernias and scrotal hernias were operated on with the TAPP than the TEP technique, a significantly higher rate of postoperative complications amenable to conservative treatment occurred in the former. These manifested as seromas, something that was consistent with these findings. Therefore, by adopting a tailored approach for inguinal hernia surgery, as recommended in the guidelines [26] on the basis of a decision-making tree [28], the indication for use of the laparoscopic technique for very large hernias and for scrotal hernias should be based on ultra stringent criteria. If the surgeon has only limited experience of the laparoscopic technique, it would be advisable to opt instead for the Lichtenstein technique in the case of a scrotal hernia with a hernia sac reaching as far as the scrotum. Only experienced TAPP experts should use laparoscopic repair for scrotal hernias. It appears that TEP surgeons are more reluctant to use this technique for scrotal hernia because of the challenging anatomic situation, indicating instead open surgical repair.

The differences in the patient collective between TEP and TAPP also explain the somewhat longer duration of operation and length of hospital stay for TAPP compared with TEP. Surgery for larger defects and a greater number of scrotal hernias result in a longer duration of operation. The significantly higher incidence of postoperative complications also leads to a longer mean length of hospital stay. However, these differences are minor and can be identified as significant only thanks to the large number of patients.

Secondary bleeding occurred significantly more often after TEP than after TAPP. In the case of TEP, the extraperitoneal space is markedly narrower than the abdominal space and impairs visibility when using current for TEP dissection. Hence, many TEP surgeons avoid the use of current and perform dissection without current, using instead a pulling and counterpulling technique, while tearing the connective tissue bridges between the anatomic structures. That inevitably results in a higher rate of secondary bleeding. Therefore, on the basis of the Herniamed data, the use current must also be recommended for dissection to reduce the secondary bleeding rate. While the use of current for dissection with the TEP technique is more onerous because of the need to clean the optics more frequently, and presumably also prolongs the duration of operation, it should be used preferably in the interest of patient risk minimization. But extreme care must be exercised when using current at the level of the peritoneum because this can cause adhesions to the intestines as well as thermal damage.

In summary, these data from the Herniamed Registry reveal that there are significant differences in perioperative outcome between TEP and TAPP. Thanks to the precise method employed for classification and for documentation of inguinal hernia defect sizes, significant differences can be identified between patients in the patient collective operated on with the TEP compared with the TAPP technique in terms of risk stratification. These differences attest to the fact that the patients operated on with the TAPP technique had significantly larger defect sizes and a significantly greater proportion of scrotal hernias. The significantly higher postoperative seroma rate in patients operated on with the TAPP technique, leading to a significantly higher overall postoperative complication rate, is therefore to be expected. To reduce the seroma rate for a directly accessible hernia, it is recommended to use widespread electrocoagulation of the pseudohernia sac for sloughing off even the smallest blood and lymph vessels or inversion of the pseudohernia sac with fixation to Cooper’s ligament [29, 30].

Consequently, the indication for TAPP for large hernia defects and for scrotal hernias should be tailored to the surgeon’s experience. Large hernias and scrotal hernias should be repaired either by a very experienced TAPP surgeon or, using a tailored approach, using the open Lichtenstein technique. Current should be employed for dissection in both TEP and TAPP operations, but extreme care must be taken to avoid secondary bleeding.

Finally, analysis of a large patient collective in routine practice has revealed that 25 years after the introduction of laparoscopic surgical techniques for inguinal hernia repair, TAPP and TEP techniques can be carried out with a very low rate of predominantly harmless complications and with an acceptable duration of operation. Today, onset of serious visceral and vascular complications is rare, even in non-specialist hospitals, but the situation is still not satisfactory. However, a further reduction can only be achieved through continuing training, accretion of knowledge and improvement of the surgical techniques.

Apart from that, assuming a comparable patient group, identical indication and adequately experienced surgeons, similar results can be achieved with the TEP and TAPP technique. That is borne out by the comparable reoperation rate for postoperative complications. The technical provisions set out in the international guidelines should continue to be observed for conduct of both TAPP and TEP [29, 30].

## Appendix: Herniamed Study Group

### Scientific Board

**Köckerling**, Ferdinand (Chairman); **Berger**, Dieter; **Bittner**, Reinhard; **Fortelny**, René; **Koch**, Andreas; **Kraft,** Barbara;**Kuthe**, Andreas; **Lorenz**, Ralph; **Mayer**, Franz; **Moesta**, Kurt Thomas; **Niebuhr**, Henning; **Peiper**, Christian; **Pross**, Matthias; **Reinpold**, Wolfgang; **Simon**, Thomas; **Stechemesser**, Bernd; **Unger**, Solveig.

### Participants

**Ahmetov**, Azat (Saint-Petersburg); **Alapatt,** Terence Francis (Frankfurt/Main); **Anders,** Stefan (Berlin); **Anderson**, Jürina (Würzburg); **Arndt**, Anatoli (Elmshorn); **Asperger,** Walter (Halle); **Avram**, Iulian (Saarbrücken); **Barkus**; Jörg (Velbert); **Becker**, Matthias (Freital); **Behrend**, Matthias (Deggendorf); **Beuleke,** Andrea (Burgwedel); **Berger,** Dieter (Baden–Baden); **Bittner,** Reinhard (Rottenburg); **Blumberg,** Claus (Lübeck); **Böckmann,** Ulrich (Papenburg); **Böhle**, Arnd Steffen (Bremen); **Böttger,** Thomas Carsten (Fürth); **Borchert,** Erika (Grevenbroich); **Born**, Henry (Leipzig); **Brabender,** Jan (Köln); **Breitenbuch von**, Philipp (Radebeul); **Brüggemann**, Armin (Kassel); **Brütting**, Alfred (Erlangen); **Budzier**, Eckhard (Meldorf); **Burghardt**, Jens (Rüdersdorf); **Carus**, Thomas (Bremen); **Cejnar**, Stephan-Alexander (München); **Chirikov**, Ruslan (Dorsten); **Comman,** Andreas (Bogen); **Crescent**i, Fabio (Verden/Aller); **Dapunt**, Emanuela (Bruneck); **Decker**, Georg (Berlin); **Demmel**, Michael (Arnsberg); **Descloux,** Alexandre (Baden); **Deusch**, Klaus-Peter (Wiesbaden); **Dick**, Marcus (Neumünster); **Dieterich**, Klaus (Ditzingen); **Dietz**, Harald (Landshut); **Dittmann**, Michael (Northeim); **Dornbusch**, Jan (Herzberg/Elster); **Drummer**, Bernhard (Forchheim); **Eckermann**, Oliver (Luckenwalde); **Eckhoff,** Jörn/Hamburg); **Elger**, Karlheinz (Germersheim); **Engelhardt**, Thomas (Erfurt); **Erichsen,** Axel (Friedrichshafen); **Eucker**, Dietmar (Bruderholz); **Fackeldey**, Volker (Kitzingen); **Farke**, Stefan (Delmenhorst); **Faust**, Hendrik (Emden); **Federmann**, Georg (Seehausen); **Feichter**, Albert (Wien); **Fiedler,** Michael (Eisenberg); **Fischer**, Ines (Wiener Neustadt); **Fortelny**, René H. (Wien); **Franczak**, Andreas (Wien); **Franke**, Claus (Düsseldorf); **Frankenberg von**, Moritz (Salem); **Frehner**, Wolfgang (Ottobeuren); **Friedhoff**, Klaus (Andernach); **Friedrich,** Jürgen (Essen); **Frings**, Wolfram (Bonn); **Fritsche**, Ralf (Darmstadt); **Frommhold,** Klaus (Coesfeld); **Frunder**, Albrecht (Tübingen); **Fuhrer**, Günther (Reutlingen); **Gassler,** Harald (Villach); **Gerdes**, Martin (Ostercappeln); **Gilg**, Kai-Uwe (Hartmannsdorf); **Glaubitz**, Martin (Neumünster); **Glutig,** Holger (Meißen); **Gmeiner**, Dietmar (Bad Dürrnberg); **Göring**, Herbert (München); **Grebe**, Werner (Rheda-Wiedenbrück); **Grothe**, Dirk (Melle); **Gürtler**, Thomas (Zürich); **Hache**, Helmer (Löbau); **Hämmerle**, Alexander (Bad Pyrmont); **Haffner**, Eugen (Hamm); **Hain**, Hans-Jürgen (Groß-Umstadt); **Hammans**, Sebastian (Lingen); **Hampe**, Carsten (Garbsen); **Harrer**, Petra (Starnberg); **Heinzmann**, Bernd (Magdeburg); **Heitland**, Tim (München); **Helbling**, Christian (Rapperswil); **Hempen**, Hans-Günther (Cloppenburg); **Henneking**, Klaus-Wilhelm (Bayreuth); **Hermes**, Wolfgang (Weyhe); **Herrgesell**, Holger (Berlin); **Herzing**, Holger Höchstadt); **Hessler**, Christian (Bingen); **Hildebrand**, Christiaan (Langenfeld); **Höferlin**, Andreas (Mainz); **Hoffmann**, Michael (Kassel; **Hofmann**, Eva M. (Frankfurt/Main); **Hopfe**r, Frank (Eggenfelden); **Hornung**, Frederic (Wolfratshausen); **Hügel**, Omar (Hannover); **Hüttemann**, Martin (Oberhausen); **Huhn**, Ulla (Berlin); **Imdahl**, Andreas (Heidenheim); **Jacob**, Dietmar (Bielefeld); **Jenert**, Burghard (Lichtenstein); **Jugenheimer**, Michael (Herrenberg); **Junger**, Marc (München); **Käs**, Stephan (Weiden); **Kahraman,** Orhan (Hamburg); **Kaiser,** Christian (Westerstede); **Kaiser**, Stefan (Kleinmachnow); **Kapischke**, Matthias (Hamburg); **Karch**, Matthias (Eichstätt); **Keck**, Heinrich (Wolfenbüttel); **Keller,** Hans W. (Bonn); **Kienzle**, Ulrich (Karlsruhe); **Kipfmüller**, Brigitte (Köthen); **Kirsch**, Ulrike (Oranienburg); **Klammer**, Frank (Ahlen); **Klatt**, Richard (Hagen); **Kleemann**, Nils (Perleberg); **Klein**, Karl-Hermann (Burbach); **Kleist**, Sven (Berlin); **Klobusicky**, Pavol (Bad Kissingen); **Kneifel**, Thomas (Datteln); **Knoop**, Michael (Frankfurt/Oder); **Knotter**, Bianca (Mannheim); **Koch,** Andreas (Cottbus); **Köckerling,** Ferdinand (Berlin); **Köhler**, Gernot (Linz); **König**, Oliver (Buchholz); **Kornblum**, Hans (Tübingen); **Krämer**, Dirk (Bad Zwischenahn); **Kraft**, Barbara (Stuttgart); **Kreissl**, Peter (Ebersberg); **Krones**, Carsten Johannes (Aachen); **Kruse,** Christinan (Aschaffenburg); **Kube**, Rainer (Cottbus); **Kühlberg**, Thomas (Berlin); **Kuhn,** Roger (Gifhorn); **Kusch,** Eduard (Gütersloh); **Kuthe,** Andreas (Hannover); **Ladberg**, Ralf (Bremen); **Ladra**, Jürgen (Düren); **Lahr-Eigen**, Rolf (Potsdam); **Lainka**, Martin (Wattenscheid); **Lammers**, Bernhard J. (Neuss); **Lancee**, Steffen (Alsfeld); **Larusson**, Hannes Jon (Pinneberg); **Lauschke**, Holger (Duisburg); **Leher,** Markus (Schärding); **Leidl**, Stefan (Waidhofen/Ybbs); **Lenz**, Stefan (Berlin); **Lesch**, Alexander (Kamp-Lintfort); **Lienert**, Mark (Duisburg); **Limberger**, Andreas (Schrobenhausen); **Locher**, Martin (Kiel); **Loghmanieh**, Siawasch (Viersen); **Lorenz**, Ralph (Berlin); **Mallmann**, Bernhard (Krefeld); **Manger**, Regina (Schwabmünchen); **Maurer**, Stephan (Münster); **Mayer**, Franz (Salzburg); **Menzel**, Ingo (Weimar); **Meurer**, Kirsten (Bochum); **Meyer**, Moritz (Ahaus**)**; **Mirow**, Lutz (Kirchberg); **Mittenzwey**, Hans-Joachim (Berlin); **Mörder-Köttgen**, Anja (Freiburg); **Moesta**, Kurt Thomas (Hannover); Moldenhauer, Ingolf (Braunschweig); **Morkramer**, Rolf (Xanten); **Mosa**, Tawfik (Merseburg); **Müller**, Hannes (Schlanders); **Münzberg**, Gregor (Berlin); **Mussack,** Thomas (St. Gallen); **Neumann**, Jürgen (Haan); **Niebuhr,** Henning (Hamburg); **Nölling**, Anke (Burbach); **Nostitz**, Friedrich Zoltán (Mühlhausen); **Obermaier**, Straubing); **Öz-Schmidt**, Meryem (Hanau); **Oldorf**, Peter (Usingen); **Olivieri**, Manuel (Pforzheim); **Pawelzik**, Marek (Hamburg); **Peiper**, Christian (Hamm); **Pertl**, Alexander (Spittal/Drau); **Philipp**, Mark (Rostock); **Pickart**, Lutz (Bad Langensalza); **Pizzera**, Christian (Graz); **Pöllath**, Martin (Sulzbach-Rosenberg); **Possin**, Ulrich (Laatzen); **Prenzel**, Klaus (Bad Neuenahr-Ahrweiler); **Pröve**, Florian (Goslar); **Pronnet**, Thomas (Fürstenfeldbruck); **Pross**, Matthias (Berlin); **Puff**, Johannes (Dinkelsbühl); **Rabl**, Anton (Passau); **Rapp**, Martin (Neunkirchen); **Reck**, Thomas (Püttlingen); **Reinpold,** Wolfgang (Hamburg); **Reuter**, Christoph (Quakenbrück); **Richter,** Jörg (Winnenden); **Riemann**, Kerstin (Alzenau-Wasserlos); **Rodehorst**, Anette (Otterndorf); **Roehr**, Thomas (Rödental); **Roncossek**, Bremerhaven); **Roth** Hartmut (Nürnberg); **Sardoschau**, Nihad (Saarbrücken); **Sauer**, Gottfried (Rüsselsheim); **Sauer**, Jörg (Arnsberg); **Seekamp**, Axel (Freiburg); **Seelig**, Matthias (Bad Soden); **Seiler**, Christoph Michael (Warendorf); **Seltmann,** Cornelia (Hachenburg); **Senkal,** Metin (Witten); **Shamiyeh**, Andreas (Linz); **Shang**, Edward (München); **Siemssen**, Björn (Berlin); **Sievers,** Dörte (Hamburg); **Silbernik**, Daniel (Bonn); **Simon**, Thomas (Sinsheim); **Sinn**, Daniel (Olpe); **Sinning**, Frank (Nürnberg); **Smaxwil**, Constatin Aurel (Stuttgart); **Schabel**, Volker (Kirchheim/Teck); **Schadd**, Peter (Euskirchen); **Schassen von**, Christian (Hamburg); **Schattenhofer**, Thomas (Vilshofen); **Scheidbach**, Hubert (Neustadt/Saale); **Schelp**, Lothar (Wuppertal); **Scherf**, Alexander (Pforzheim); **Scheyer**, Mathias (Bludenz); **Schimmelpenning,** Hendrik (Neustadt in Holstein); **Schinkel**, Svenja (Kempten); **Schmid**, Michael (Gera); **Schmid,** Thomas (Innsbruck); **Schmidt**, Rainer (Paderborn); **Schmidt**, Sven-Christian (Berlin); **Schmidt,** Ulf (Mechernich); **Schmitz**, Heiner (Jena); **Schmitz**, Ronald (Altenburg); **Schöche**, Jan (Borna); **Schoenen**, Detlef (Schwandorf); **Schrittwieser**, Rudolf/Bruck an der Mur); **Schroll**, Andreas (München); **Schultz**, Christian (Bremen-Lesum); **Schultz**, Harald (Landstuhl); **Schulze**, Frank P. Mülheim an der Ruhr); **Schumacher**, Franz-Josef (Oberhausen); **Schwab**, Robert (Koblenz); **Schwandner**, Thilo (Lich); **Schwarz**, Jochen Günter (Rottenburg); **Schymatzek**, Ulrich (Radevormwald); **Spangenberger**, Wolfgang (Bergisch-Gladbach); **Sperling**, Peter (Montabaur); **Staade**, Katja (Düsseldorf); **Staib**, Ludger (Esslingen); **Stamm**, Ingrid (Heppenheim); **Stark**, Wolfgang (Roth); **Stechemesser,** Bernd (Köln); **Steinhilper**, Uz (München); **Stern**, Oliver (Hamburg); **Stolte**, Thomas (Mannheim); **Stopinski,** Jürgen (Schwalmstadt); **Stubbe**, Hendrik (Güstrow/); **Stülzebach**, Carsten (Friedrichroda); **Tepel,** Jürgen (Osnabrück); **Terzić**, Alexander (Wildeshausen); **Teske,** Ulrich (Essen); **Thews**, Andreas (Schönebeck); **Tillenburg**, Wolfgang (Marktheidenfeld); **Timmermann,** Wolfgang (Hagen); **Train**, Stefan H. (Gronau); **Trauzettel**, Uwe (Plettenberg); **Triechelt**, Uwe (Langenhagen); **Ulcar**, Heimo (Schwarzach im Pongau); **Unger**, Solveig (Chemnitz); **Verweel**, Rainer (Hürth); **Vogel**, Ulrike (Berlin); **Voigt**, Rigo (Altenburg); **Voit**, Gerhard (Fürth); **Volkers**, Hans-Uwe (Norden); **Vossough**, Alexander (Neuss); **Wallasch**, Andreas (Menden); **Wallner**, Axel (Lüdinghausen); **Warscher,** Manfred (Lienz); **Warwas**, Markus (Bonn); **Weber**, Jörg (Köln); **Weiß**, Johannes (Schwetzingen); **Weißenbach**, Peter (Neunkirchen); **Werner**, Uwe (Lübbecke-Rahden); **Wessel,** Ina (Duisburg); **Weyhe**, Dirk (Oldenburg); **Wieber**, Isabell (Köln); **Wiesmann**, Aloys (Rheine); **Wiesner**, Ingo (Halle); **Woehe**, Fritz (Sanderhausen); **Wolf**, Claudio (Neuwied); **Yildirim**, Selcuk (Berlin); **Zarras**, Konstantinos (Düsseldorf); **Zeller**, Johannes (Waldshut-Tiengen); **Zhorzel**, Sven (Agatharied); **Zuz**, Gerhard (Leipzig);
